# Unveiling common psychological characteristics of proneness to aggression and general psychopathology in a large community youth cohort

**DOI:** 10.1038/s41398-023-02538-8

**Published:** 2023-07-12

**Authors:** Ting Yat Wong, Zhiqian Fang, Charlton Cheung, Corine S. M. Wong, Yi Nam Suen, Christy L. M. Hui, Edwin H. M. Lee, Simon S. Y. Lui, Sherry K. W. Chan, Wing Chung Chang, Pak Chung Sham, Eric Y. H. Chen

**Affiliations:** 1 Department of Psychiatry, Perelman School of Medicine, University of Pennsylvania, Philadelphia, USA; 2 Department of Psychiatry, The University of Hong Kong, Hong Kong SAR, China; 3 Department of Psychology, Education University of Hong Kong, Hong Kong SAR, China; 4 State Key Laboratory of Brain and Cognitive Sciences, University of Hong Kong, Hong Kong SAR, China

**Keywords:** Psychiatric disorders, Human behaviour

## Abstract

Elevated aggression in individuals with psychiatric disorders is frequently reported yet aggressive acts among people with mental illness are often intertwined with proneness to aggression and other risk factors. Evidence has suggested that both general psychopathology and proneness to aggression may share common psychological characteristics. This study aims to investigate the complex relationship between general psychopathology, proneness to aggression, and their contributing factors in community youth. Here, we first examined the association between proneness to aggression and the level of general psychopathology in 2184 community youths (male: 41.2%). To identify common characteristics, we trained machine learning models using LASSO based on 230 features covering sociodemographic, cognitive functions, lifestyle, well-being, and psychological characteristics to predict levels of general psychopathology and proneness to aggression. A subsequent Gaussian Graph Model (GGM) was fitted to understand the relationships between the general psychopathology, proneness to aggression, and selected features. We showed that proneness to aggression was associated with a higher level of general psychopathology (discovery: *r* = 0.56, 95% CI: [0.52–0.59]; holdout: *r* = 0.60, 95% CI: [0.54–0.65]). The LASSO model trained on the discovery dataset for general psychopathology was able to predict proneness to aggression in the holdout dataset with a moderate correlation coefficient of 0.606. Similarly, the model trained on the proneness to aggression in the discovery dataset was able to predict general psychopathology in the holdout dataset with a correlation coefficient of 0.717. These results suggest that there is substantial shared information between the two outcomes. The GGM model revealed that isolation and impulsivity factors were directly associated with both general psychopathology and proneness to aggression. These results revealed shared psychological characteristics of general psychopathology and proneness to aggression in a community sample of youths.

## Introduction

Extensive research has investigated the relationship between aggression and general mental health. Although aggression is generally considered a core symptom of externalizing disorders, studies have found that it is often observed among individuals with other severe mental illnesses, including depression, bipolar disorders, and schizophrenia spectrum disorders [1, 2]. A recent review also noted that previous studies have identified psychiatric diagnosis as a risk factor for aggression [3]. In contrast, youths who are prone to aggression may experience compromised general mental health, with an elevated risk of experiencing internalizing symptoms such as anxiety [4], emotional eating [5], hoarding [6], and psychotic-like experiences [7]. Moreover, high proneness to aggression often increases the likelihood of aggressive actions under provocation [8], and individuals with psychiatric disorders who have a higher proneness to aggression are more likely to exhibit aggressive behavior [9, 10]. While previous studies have identified the relationship between aggression and mental health, little is known about the underlying factors that may contribute to the co-occurrence of proneness to aggression and mental health symptoms, particularly in a nonclinical youth population.

Aggression was found to be associated with common psychiatric conditions and strongly linked to the total number of lifetime psychiatric disorders [11]. This suggests that aggression may not be merely a symptom of specific mental health disorders, but rather a more general marker of overall psychopathology. A recent study utilizing dimensional models of psychopathology has shown that the transdiagnostic composite of internalizing symptoms is strongly associated with proneness to aggression [12], highlighting the importance of understanding the relevance of proneness to aggression in the context of transdiagnostic psychopathology symptoms. However, despite these advancements, there is a lack of research considering the construct of general psychopathology that encompasses symptoms from multiple mental disorders. By identifying the shared underlying mechanisms that contribute to both general psychopathology and proneness to aggression, we can gain a more comprehensive understanding of the intricate interplay between these constructs.

Transdiagnostic factors such as impulsivity, top-down cognitive control, emotion regulation, negative affectivity, and neurotic/antagonistic personality, have been found to be associated with both general psychopathology [13–15] and proneness to aggression [16–18] in youth. These findings suggest that general psychopathology and proneness to aggression may share underlying factors. These contributing factors may also be related to each other, adding complexity to their relationship with proneness to aggression and psychopathology. Additionally, neuroimaging studies have revealed overlapping structural and functional abnormalities involved in cognitive control, social functioning, and emotion processing for proneness to aggression [19, 20]. Prior meta-analysis has identified disrupted brain networks implicated in executive functioning, action inhibition, and higher-level cognition across psychiatric disorders [21]. Although these neuroimaging findings were not specific to youth, they still provide support for the possibility of shared underlying mechanisms between these constructs. However, further research is needed to understand whether, and more importantly what, psychological characteristics are linking these constructs together [22]. Although available evidence suggests a potential overlap in contributing factors, no study has systematically examined the relationships between proneness to aggression, psychopathology, and contributing factors together while controlling for the dynamic relationships among the variables.

Adolescence and youth are critical developmental stages marked by significant changes in behavior, cognition, and emotion as individuals transition from childhood to adulthood [23, 24]. During this period, both psychopathology and aggression are prevalent [25–29]. Aggression can have serious negative consequences for both individuals and society [30]. Understanding the common contributing factors to both general psychopathology and proneness to aggression during this period could provide a clearer picture of the development of aggressive behaviors. Besides, it is well-established that mental health problems (e.g., internalizing and externalizing problems) are often associated with proneness to aggression and can exacerbate aggressive behavior [31–34]. Recognizing the complex dynamics between proneness to aggression, general psychopathology, and their contributing factors can inform strategies for (1) preventing escalating proneness to aggression into actual aggressive behaviors during the transition from youth to adulthood, (2) tailoring interventions to address the specific factors that contribute to both aggression and general mental wellness, and (3) reducing the stigma towards violence in mental health.

The primary aim of the study is to investigate the complex interplay between general psychopathology, proneness to aggression, and their contributing factors to identify common factors associated with both phenomena. We first established the relationship between general psychopathology and proneness to aggression in a community nonclinical youth cohort. A population-based epidemiological study in Hong Kong measured a wide range of domains, including sociodemographics, cognitive functions, lifestyle, well-being, and psychological characteristics. This provides a great opportunity to identify common characteristics between general psychopathology and proneness to aggression in youths through a data-driven approach. By applying a machine learning approach, we aim to demonstrate that proneness to aggression is robustly associated with a higher level of general psychopathology in the nonclinical youth population. A set of overlapping features was expected across prediction models for both phenomena. In addition, we examined the cross-prediction accuracy of trained models and hypothesized that a trained prediction model for general psychopathology could predict proneness to aggression and vice versa. To gain a deeper understanding of the complex relationship between overlapping features and outcomes (i.e., general psychopathology and proneness to aggression), while accounting for the influence of other intercorrelated variables, we constructed a Gaussian Graph Model (GGM). Based on prior research and limited by our own variables, we expected that impulsivity, top-down cognitive control, and neurotic/antagonistic personality factors would merge to be the shared features linked to both general psychopathology and proneness to aggression [35, 36].

## Methods

### Epidemiological samples

The Hong Kong Youth Epidemiological Study (HKYES) cohort aged between 15 and 24 has been recruited using a multistage stratified sampling design since 2016 (https://www.hkyes.hku.hk). The current study analyzed a subset of the participants from this ongoing project using relevant items to answer our specific research questions. At the time of the query (21 June 2021), a total of 2544 youths were surveyed. Consent or assent was obtained from all participants. All interviews were conducted using computer-assisted personal interviewing [37] with the Qualtrics survey platform (Qualtrics, Provo, UT, USA), in which research assistants assist the administration of the interview, and participants can read and answer the questions themselves. Monetary compensation was offered to participants upon completion of the study. Ethical approval was obtained from the local review board.

### Outcomes: psychopathology and proneness to aggression

This study is a secondary data analysis as the HKYES project does not especially aim to answer the current research questions. The research questions and hypotheses were formulated during data collection. Data analysis was conducted from September 2021 to March 2022. Proneness to aggression was measured by the sum of the 12-item short form of the Buss-Perry Aggression Questionnaire (BPAQ) [38, 39]. The BPAQ can be further explained by four intercorrelated factors, including physical aggression, verbal aggression, anger, and hostility [39]. Individual levels of general psychopathology were defined as the first principal component across all dimensional measures of symptom levels for common mental illness, including depression, mania, hypomania, general anxiety, social anxiety, obsessive–compulsive symptoms, psychotic-like experiences, and prodromal psychotic symptoms. The use of the first principal component analysis (PCA) component as a measure of general psychopathology is a widely accepted and valid approach [40]. Details of these scales can be referred to in the Supplementary materials (Table S1).

### Features

We included a total of 300 items from 29 scales (see Table S2 for details) as features for predicting general psychopathology and proneness to aggression. None of these features directly measure any construct representing proneness to aggression or psychopathology. These items reflect each participant’s sociodemographic (sex, age, body mass index, education), cognitive functioning, lifestyle (sleep, physical activity, body perception, drinking), general well-being, and psychological characteristics (personality, self-esteem, impulsivity, sensation seeking, future outlook, goal commitment, procrastination, resilience, prosocial behavior, materialism, and loneliness).

### Data preprocessing

Individual item-wise features with more than 25% missing were removed. Items using string as a response were excluded. Therefore, 230 features remained for the subsequent analyses. A total of 360 participants were excluded since they had missing values in any individual items of the outcome measures, age, sex, and/or more than 5% missing values out of all the items.

### Correlation between psychopathology and proneness to aggression

Pearson’s correlations between proneness to aggression and general psychopathology were established in the discovery and holdout samples separately. Correlations between subfactors of BPAQ and individual symptom scales were also calculated for further interpretation. The false discovery rate (FDR) approach was used to adjust the *p*-values for multiple comparisons with a threshold of *q*-values < 0.05 [41].

### Prediction models for general psychopathology and proneness to aggression

Prediction models were trained using a LASSO regression to examine robust relationships among the outcomes. The LASSO is an L1-norm regularized regression model approach that retains only the most significant variables and tends to remove unnecessary ones by forcing their regression coefficients to zero [42]. Thus, this procedure allows us to keep the most important individual items in predicting the outcomes for the subsequent network analyses.

**Fig. 1:**
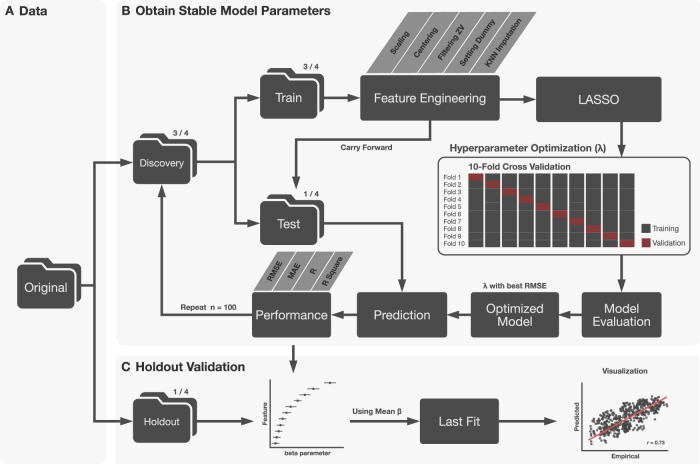
Schematic workflow of the LASSO model. **A** The original data were split into discovery (3/4) and holdout (1/4) samples. **B** The discovery samples were further divided into train (3/4) and test (1/4) samples. In each iteration, the train samples were preprocessed and fitted to obtain an optimal hyperparameter using root mean square error (RMSE) for the LASSO model using 10-fold cross-validation. The optimized model was fitted to the test samples and model performance and the beta parameters were stored. These procedures were repeated 100 times. **C** 95% confidence intervals (CIs) of feature beta parameters were calculated. Features in which 95% of CIs did not cross zero were considered significant and the rest were set to zero. We averaged beta parameters of significant features and validated the performance in the holdout samples.

The schematic workflow of the LASSO model is illustrated in Fig. 1. We first divided the dataset into two portions: the third-fourth of the data were randomly selected as a discovery dataset and the rest were used as a holdout dataset. For the discovery dataset, we further split a third-fourth of the data into the “train” dataset and one-fourth into the “test” dataset. The training dataset was used for hyperparameter tuning and training an optimized model while the test dataset was used for testing the optimized model with the best penalty parameter obtained from training. All the numerical features and outcomes were scaled and centered, and all nominal data were transformed into a dummy variable (i.e., yes and no for each category). Features with zero variance (i.e., the same response across all participants) were excluded from the pipeline. Missing data were imputed with k-nearest-neighbors (*k* = 20). The above procedures were performed on the training data and carried forward on the testing data. Preprocessed training data were fitted to a LASSO model with a 10-fold cross-validation procedure with a *λ* (the penalty factor) ranging from 1e^−^^10^ to 1. The best λ was selected based on the root mean square error (RMSE). The optimized model was fitted using the training data for predicting general psychopathology and proneness to aggression in the unseen testing data. The performance of the optimized model was examined by the RMSE. This random subsampling procedure was repeated 100 times. For each iteration, the best λ value, the performance metrics including RMSE, mean absolute error (MAE), accuracy (i.e., the correlation between empirical and predicted scores), and *R*^2^, as well as the parameter importance and beta parameters of the LASSO models, were recorded. The mean beta parameters across 100 models were used to predict the outcomes in the holdout dataset (Fig. 1C). Please note that only features whose 95% confidence intervals across 100 models did not cross zero were considered significant features. The beta parameters of insignificant features were set to zero. Next, we extracted the overlapping non-zero (i.e., significant) features across two prediction models. Cross-predictions were performed to investigate if the trained model for general psychopathology can be used to predict proneness to aggression and if the model for proneness to aggression can be used to predict general psychopathology. This procedure can determine if these two outcomes shared common information.

We have replaced LASSO with Elastic Net and repeated the same prediction model workflow for a sensitivity evaluation. The Elastic Net model allows us to handle high-dimensional data with potential collinearity among the predictors with the advantage of combining the L1 and L2 regularization techniques [43]. We have included the details of the Elastic Net method in the supplementary materials.

### Understanding dynamics between overlapping features and outcomes

Network analyses further explored the relationships between outcomes and features. For this purpose, we used the samples without any missing values (complete dataset; *n* = 1673). Table S3 shows that there was no difference between the original dataset and the complete dataset.

Dimensions of overlapping non-zero features were further reduced by applying a factor analysis using minimum residual and “oblimin” rotation. “Oblimin” rotation allows the factors to not be orthogonal as for the subsequent exploratory network analyses using partial correlations. The parallel analysis was performed to determine the optimal number of factors as the features for the network analyses [44].

A Gaussian Graphical Model (GGM) was fitted. In a GGM network, each node represents an item from features and outcomes. An edge between any two nodes indicates a partial correlation between two variables, after adjusting for all other variables in the dataset. Due to the large sample size of the current study (*n* > 1000), an unregularized model selection was used in estimating GGMs [45] using the *R*-package *qgraph* (version 1.9.1) function *ggmModSelect* [46]. The constructed network is visualized with a spring layout [47]. Blue (red) edges in the network graph indicate positive (negative) partial correlations and the stronger partial correlations are represented by a wider and more saturated edge [46]. The expected influence was calculated as the centrality measure. The stability of networks was examined by estimating the stability of edge strengths via nonparametric bootstrapping and expected influence using case-drop bootstrapping via *R*-package *bootnet* (version 1.5) [48, 49].

All the inputs were transformed using the *R*-package *bestNormalize* (version 1.8.3) to ensure normality [50]. The *bestNormalize* function estimates the optimal normalizing transformation on the basis of the Pearson P test statistic for normality [50]. The transformation method includes: no transformation, the Yeo–Johnson transformation, the Box–Cox transformation (if the data is positive), the log_10_(x + a) transformation, the square-root (*x* + *a*) transformation, the arcsinh transformation, and ordered quantile normalization [51]. The transformation method with the lowest Pearson *P* test statistic was selected. This automated procedure was able to transform the input variables to approximate normality.

To display how the outcomes are related to the features, we computed flow diagrams using the *flow* function from *qgraph*. A flow diagram takes one node as a source and lists all the nodes that have an edge with the source node and how these nodes connect to the rest of the nodes.

## Results

### Participants

Numerical values with ± represent a mean and its standard deviation. A total of 2184 youths (male: *n* = 899, age = 19.8 ± 2.83 years; female: *n* = 1285, age = 20.0 ± 2.75 years) were included after applying the exclusion criteria. Table 1 shows that age, gender, years of education, proneness to aggression, and general psychopathology were similar in the discovery (*n* = 1638) and holdout (*n* = 546) datasets (Table 1, *ps* > 0.05).

**Table 1:** Summary statistics of the discovery and holdout samples.

Characteristic	Overall, *N* = 2184^a^	Discovery, *N* = 1638^a^	Holdout, *N* = 546^a^	*p*-Value^b^	*q*-Value^c^
Age	19.93 (2.78)	19.92 (2.75)	19.96 (2.87)	0.7	>0.9
Sex				0.5	>0.9
Males	899 (41%)	667 (41%)	232 (42%)		
Females	1285 (59%)	971 (59%)	314 (58%)		
Years of education	13.42 (2.47)	13.42 (2.44)	13.44 (2.55)	0.7	>0.9
Proneness to aggression	19.58 (6.52)	19.55 (6.48)	19.66 (6.65)	>0.9	>0.9
General psychopathology	0.01 (1.68)	0.00 (1.67)	0.05 (1.73)	0.7	>0.9

No significant difference between these two samples was found.

^a^Mean (SD); *n* (%).

^b^Wilcoxon rank sum test; Pearson’s Chi-squared test.

^c^False discovery rate correction for multiple testing.

### Proneness to aggression was associated with higher levels of general psychopathology

**Fig. 2:**
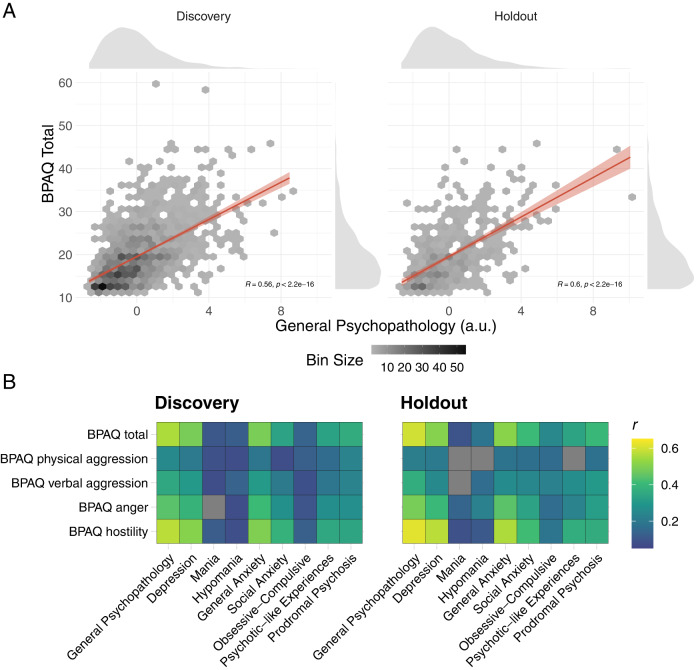
Pearson correlations between psychopathology and proneness to aggression. **A** Hexagon plots show that proneness to aggression was associated with a higher level of general psychopathology in the discovery and holdout datasets. The saturation represents the number of samples within each bin. **B** The heat map further shows the correlations between individual measures of psychopathology symptoms and subfactors of proneness to aggression. Gray cells indicate that correlations do not pass the threshold (i.e., *q*s > 0.05).

The first PCA component across psychopathology scales representing the general psychopathology explained 34.8% variances and all psychopathology scales were positively loaded to this component (Fig. S1). Figure 2A shows that proneness to aggression was associated with a higher level of general psychopathology in the discovery (*r* = 0.56, 95% CI: [0.52–0.59], *p* < 0.001) and holdout (*r* = 0.60, 95% CI: [0.54–0.65], *p* < 0.001) datasets. Figure 2B further shows the correlations between specific BPAQ factors and symptom levels of individual psychopathology. Insignificant correlations with subfactors of proneness to aggression were found in mania, hypomania, and psychotic-like symptoms. The rest of the associations were statistically significant after controlling for multiple comparisons using FDR (*q*s < 0.05).

### General psychopathology and proneness to aggression share common psychological characteristics

**Fig. 3:**
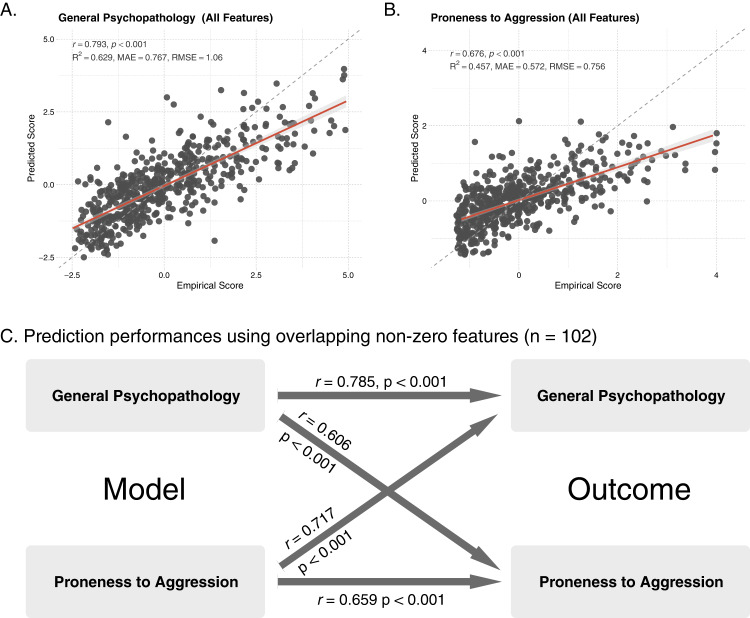
Performance for predicting general psychopathology and proneness to aggression. **A**, **B** Using all features, the accuracies for predicting general psychopathology and proneness to aggression were moderate to high. **C** Models using only overlapping non-zero features performed as well as models using all features. The trained model for proneness to aggression can accurately predict general psychopathology (*r* = 0.717). The trained model for the general psychopathology model can accurately predict proneness to aggression (*r* = 0.606).

LASSO models revealed 141 significant (i.e., non-zero) features for predicting general psychopathology and 157 significant features for predicting proneness to aggression across 100 random subsampling procedures. The averaged beta parameters were applied to the holdout dataset. Correlations between empirical and predicted scores of general psychopathology and proneness to aggression were calculated to evaluate prediction performance. Figure 3A, B show that the prediction accuracies in predicting general psychopathology (*r* = 0.793, *p* < 0.001, *R*^2^ = 0.629, MAE = 0.767, RMSE = 1.06) and proneness to aggression (*r* = 0.676, *p* < 0.001, *R*^2^ = 0.457, MAE = 0.572, RMSE = 0.756) were moderate to good. Furthermore, 102 overlapping non-zero features across models were identified and used in the cross-predictions. Compared to models with all features, Fig. 3C shows that the prediction models with only overlapping features achieve a similar level of performance (general psychopathology: *r* = 0.785, *p* < 0.001, *R*^2^ = 0.616, MAE = 0.801, RMSE = 1.11; proneness to aggression: *r* = 0.659, *p* < 0.001, *R*^2^ = 0.434, MAE = 0.587, RMSE = 0.784). Using parameters from the proneness to aggression model to predict general psychopathology, the accuracy was *r* = 0.717 (*p* < 0.001, *R*^2^ = 0.514, MAE = 1.05, RMSE = 1.39). Using parameters from the psychopathology model to predict proneness to aggression, the correlation was *r* = 0.606 (*p* < 0.001, *R*^2^ = 0.367, MAE = 0.712, RMSE = 0.922). We have repeated the same workflow using Elastic Net (see supplementary methods and results). The performance of the Elastic Net models was comparable to the LASSO models (Fig. S2). However, when it comes to cross-prediction performance, LASSO models outperformed the Elastic Net. Since our main objective is to comprehend the common features of general psychopathology and proneness to aggression, LASSO is still the preferred choice.

### Dynamics between the outcome and features

**Fig. 4:**
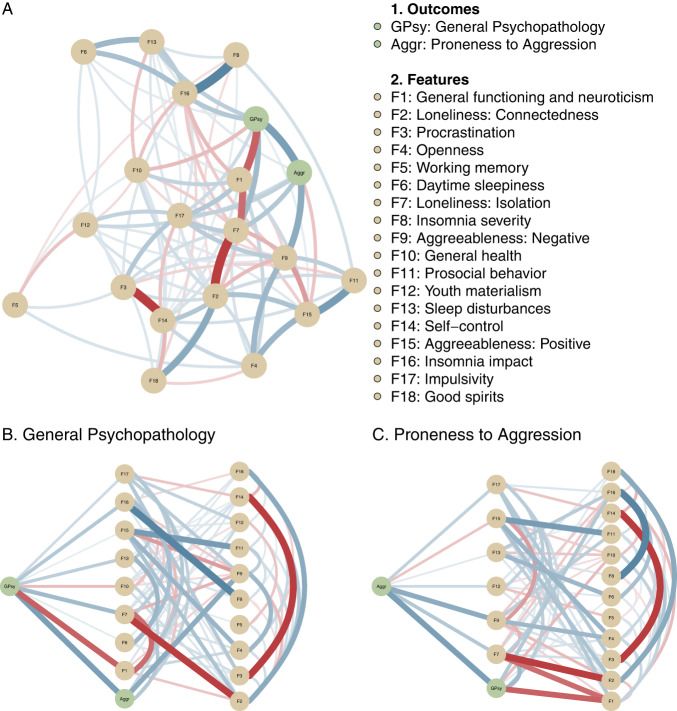
The interactions between outcomes (green nodes) and features (yellow nodes) estimated by GGM. **A** The blue edges indicate a positive effect between a pair of nodes while the red edges indicate a negative effect. The wider and more saturated color of the edge, the stronger connection is. **B**, **C**. Flow diagrams show connectivity from the general psychopathology and proneness to aggression.

Parallel analysis suggested 18 factors over 102 overlapping features (Fig. S3). The correlations and loadings of each factor can be found in Fig. S4. Histogram plots of the transformed variables were shown in Fig. S5. Figure 4A shows the estimated GGM of the 18 factors (yellow nodes) and outcomes (green nodes). The central node was F17 (Impulsivity, Fig. S6). To zoom in on the results, Fig. 4B, C shows the shortest connections from the outcomes. We revealed that three factors were positively associated with both outcomes, including F7 (Loneliness: Isolation), F13 (Sleep disturbances), and F17 (Impulsivity). However, bootstrapped stability of edge strengths revealed that only connections from F7 or F17 to the outcomes were stable (Table 2, Fig. S7). In addition, the strength centrality estimate was stable, with a centrality stability coefficient of 0.85, indicating that 75% of the data could be dropped to retain with 95% certainty a correlation of 0.7 with the original dataset (Fig. S8) [48].

**Table 2:** The nonparametric bootstrapping analysis shows the stability of connected nodes between features and outcomes.

		Edge strength		
Node1	Node2	Sample	Bootstrapped mean	Bootstrapped 95% CI
GPsy	F7	0.208	0.200	0.151, 0.250
Aggr	F7	0.173	0.167	0.114, 0.220
GPsy	F13	0.183	0.181	0.127, 0.235
Aggr	F13	0.0676	0.035	−0.045, 0.115
GPsy	F17	0.118	0.114	0.085, 0.179
Aggr	F17	0.135	0.144	0.114, 0.204

*GPsy* general psychopathology, *Aggr* proneness to aggression, *F7* loneliness-isolation, *F13* sleep disturbances, *F17* impulsivity, *CI* confidence intervals.

## Discussion

A moderate but robust positive correlation between general psychopathology and proneness to aggression was found in this large epidemiological youth cohort, consistent with another study in children that reported similar results [4, 12]. It is noteworthy that the general psychopathology factor assessed in the current study does not reflect any externalizing psychopathology due to the study design, highlighting the potential transdiagnostic nature of proneness to aggression in non-externalizing disorders. Leveraging machine learning and network approaches with 230 behavioral features, our cross-prediction results showed that a trained prediction model for proneness to aggression can be used to predict psychopathology and vice versa, suggesting that these two phenomena contained substantial shared information. Network models further explored the dynamics between the outcomes and features and revealed that both general psychopathology and proneness to aggression were directly associated with impulsivity and isolation. Our findings indicate that proneness to aggression and general psychopathology were correlated with distinct yet overlapping features, suggesting that these constructs are interrelated but still separate entities.

The period of youth is a critical phase of development for the affect and reward system, characterized by heightened stress due to the gradual transition from childhood to adulthood [52–54]. Consistent with existing literature, our research indicates that impulsivity is a common psychological attribute in both general psychopathology and proneness to aggression among young individuals [35, 55–57]. The impulsivity factor identified in our study was mainly composed of attentional and motor impulsiveness items [58]. In particular, the GGM model showed that the impulsivity factor was the key variable with high centrality in the network. Notably, a self-control factor (F14) with items related to non-planning and other top-down cognitive control was not directly linked to both proneness to aggression and general psychopathology. The earlier literature suggested that the mismatched development between developing prefrontal systems for top-down control and the relatively more mature limbic structures could enhance affective and incentive-based behaviors [52]. A recent meta-analysis has demonstrated robust delay discounting in eight common psychiatric disorders, including schizophrenia and depression, which supports the notion that impulsivity is a core transdiagnostic trait of psychiatric disorders [35, 59–61]. Our findings extend this relationship further to suggest that impulsivity may be a factor explaining the association between general psychopathology and proneness to aggression in youth. Impulsivity may lead to risky decision-making [62] and poor self-regulation [63], which in turn may increase the risk of developing psychopathology and proneness to aggression. As increased impulsivity is frequently reported during adolescence [35], our research highlights the importance of impulsivity as a common feature of psychopathology and proneness to aggression. Thus, impulsivity could be a target for prevention and intervention strategies for reducing the risk of developing psychopathology and proneness to aggression among youths.

Loneliness, especially isolation, has long been recognized as a risk factor for the mental health of children and adolescents [64, 65]. Lonely individuals tended to respond more aggressively and loneliness was associated with aggressive attitudes and hostility [66]. More importantly, early life social experiences shape neurodevelopment. In mice models, social isolation during adolescence disrupted orbitofrontal development and decision-making [67]. Our results also showed that both general psychopathology and proneness to aggression were strongly linked to isolation from the loneliness scale. Isolation together with impulsivity and depression increase the likelihood of aggressive acts in inpatients compared to those without these factors [68]. These findings supported that isolation could be a potential common factor for both psychopathology and proneness to aggression.

Psychopathology may elevate the risk of aggressive behaviors [69]. Individuals with high proneness to aggression were prompt to act aggressively under provocation while the effect of proneness to aggression on aggressive behavior could be reduced by situational inhibition [16]. Furthermore, evidence shows that the positive associations between severe mental illness and aggressive behaviors were not preserved after controlling for impedance (including proneness to aggression) and disinhibition [70]. Given that psychopathology was also positively associated with proneness to aggression and they share common psychological characteristics, it is possible that proneness to aggression influences aggressive behaviors more directly while associations between psychopathology and aggressive behavior could be overestimated. Thus, targeting only psychopathology or psychiatric diagnosis as a prevention strategy for aggressive behaviors or violence may have limited impacts [70]. Instead, confounders like impulsivity and isolation among psychopathology and proneness to aggression could be potential targets for preventing aggressive behaviors. Note that only community youths were recruited in the current study so the results should be interpreted with caution. Future studies are needed to examine whether our results can be generalized to a psychiatric population.

Several limitations should be acknowledged. First, although we have examined the effect of a wide range of factors including sociodemographic, lifestyle, cognition, and psychological constructs on proneness to aggression and general psychopathology, some risk factors may still not be fully captured. The current methodological approach restricts the maximum number and domains of features we can collect. Future studies could incorporate passive data collection in which information can be collected without the involvement of the person being observed. Second, the lack of an external validation sample may limit the generalizability of our results. Given the uniqueness of the current sample, we have randomly split our data into discovery and holdout datasets. Third, due to the cross-sectional epidemiological design, it is difficult to determine cause and effect of variables. Thus, causes and effects among variables cannot be established. Future experimental or longitudinal studies could attempt to unveil the causal effects among the variables. Fourth, GGM assumes that the input variables follow a Gaussian distribution. Despite the application of a robust transformation, it was observed that a few variables, such as proneness to aggression, were only approximately normally distributed. Fifth, LASSO may select only one/a few highly correlated (i.e., collinear) variables due to its regularization property [42, 43]. Although we have repeated the procedure 100 times and used averaged beta-coefficients across repeats to increase stability in the selected variables, it is still possible that some important variables may be excluded. Lastly, adopting a holistic approach to psychopathology provides a more comprehensive understanding of mental health while identifying commonalities and underlying mechanisms that may span across various mental health symptoms. This broader perspective enables the implementation of interventions targeting common underlying factors contributing to aggression. Proneness to aggression may emerge as a pertinent clinical concern with implications for treatment planning and early intervention strategies, particularly among individuals seeking mental health services who exhibit high levels of impulsivity and social isolation. Understanding and addressing these traits becomes crucial in developing effective strategies to prevent future aggressive behaviors. However, relying solely on general psychopathology may overlook the unique characteristics associated with individual psychiatric symptom dimensions. Hence, future studies should examine both the shared and distinct associations between psychopathology and aggression to tailor treatment interventions according to individual needs and vulnerabilities.

In summary, our study provides evidence of a robust association between general psychopathology and proneness to aggression in a large nonclinical youth cohort. This finding highlights the importance of considering the interplay between these two constructs in future mental health research. Our study also contributes to a more comprehensive understanding of the psychological factors that are associated with both general psychopathology and proneness to aggression. Specifically, we identified overlapping features including impulsivity and isolation that could have implications for the development of prevention and intervention strategies for aggression in youth with mental health issues. By targeting these common factors directly, we may be able to reduce the risk of aggression and promote better mental health outcomes in this population.

## Supplementary information

supplemental material

## Data Availability

The analysis scripts for the current study are publicly accessible via a Github repository https://github.com/kamione/yes_youth_aggression.
