# Supplementary material for: Unveiling common psychological characteristics of proneness to aggression and general psychopathology in a large community youth cohort
Source: Transl Psychiatry. 2023 Jul 12;13:255. doi: 10.1038/s41398-023-02538-8 (PMC10338447; doi:10.1038/s41398-023-02538-8)
Supplement: Supplementary file 1 — supplemental material [file 41398_2023_2538_MOESM1_ESM.docx]

**Unveiling common psychological characteristics of proneness to aggression and general psychopathology in a large community youth cohort**

Ting Yat Wong, Zhiqian Fang, Charlton Cheung, Yi Nam Suen, Christy LM Hui, Sherry KW Chan, Edwin HM Lee, Simon SY Lui, Corine SM Wong, Wing Chung Chang, Pak Chung Sham, Eric YH Chen

**Supplementary Materials**

- **Methods**
- **Results**
- **Table S1. Dimensional scales included in the general psychopathology construct with references.**
- **Table S2. The 29 scales with 300 items were included as predicting features of general psychopathology and aggression with references.**
- **Table S3. No difference between the original dataset and complete samples was found.**
- **Figure S1. Results of the PCA analysis on symptom levels of psychopathology.**
- **Figure S2. Performance for predicting general psychopathology and proneness to aggression using Elastic Net.**
- **Figure S3. Parallel analysis suggested 18 latent factors over 102 overlapping features.**
- **Figure S4. Results of PCA analysis on the overlapping features of general psychopathology and proneness to aggression.**
- **Figure S5. Histogram plots of transformed input variables of the GGM.**
- **Figure S6. Node centrality indices of the GGM network.**
- **Figure S7. Stability of edge strengths.**
- **Figure S8. Stability of centrality index.**

**Methods**

***Elastic Net Prediction Models for General Psychopathology and Proneness to Aggression***

Our objective was to streamline the selection of items by minimizing the number of features, which is why we chose to use LASSO. For this purpose, it is essential to have some coefficients shrink to zero. While Ridge regression can provide parameter estimates, it may result in biased estimates and does not necessarily reduce the number of features. We also implemented an Elastic Net regression, which combines the advantages of both LASSO (L1 penalization) and Ridge (L2 penalization). This approach enables us to simultaneously shrink some coefficients to zero while also providing parameter estimates for the remaining features. A mixture hyperparameter (from 0 to 1) for Elastic Net represents the proportion of L1 or L2 and it was added in training.

**Results**

***Elastic Net***

The out-of-sample performance of Elastic Net regression is comparable to that of LASSO regression, based on the hold-out validation results (**Figure S2**). We also observed that when more overlapping non-zero features were included (n = 169, including 99/102 features selected from LASSO), the performance of Elastic Net models for predicting outcomes using all features or overlapping non-zero features was essentially the same. However, the cross-prediction performance of the Elastic Net model was not as good as that of the LASSO model. This could suggest that the inclusion of more features in the Elastic Net model may introduce additional noise when predicting the other outcome (i.e., the general psychopathology model predicting proneness to aggression or vice versa). As our study aims to identify common psychological characteristics of general psychopathology and proneness to aggression, the additional analyses indicate that LASSO regression remains the optimal choice.

**Table S1. Dimensional scales included in the general psychopathology construct with references.**

| **Scales** | **Remarks** |  |
| --- | --- | --- |
| Patient Health Questionnaire (PHQ-9) (1) | severity of depression severity |  |
| Young Mania Rating Scale (YMRS) (2) | mania symptoms |  |
| Hypomania Checklist (HCL-32) (3) | hypomanic symptoms |  |
| Generalized Anxiety Disorder scale (GAD-7) (4) | the severity of generalized anxiety disorder |  |
| Liebowitz Social Anxiety Scale (LSAS) (5) | Symptom severity of social phobia |  |
| Yale-Brown Obsessive Compulsive Scale (Y-BOCS) (6) | Symptom severity of common obsessions and compulsions |  |
| Community Assessment of Psychic Experiences positive symptom scale (CAPE-P-15) (7) | Persecutory ideation, bizarre experiences, and perceptual anomalies |  |
| Prodromal Questionnaire–Brief Version (PQ-B) (8) | Screening measure for psychosis risk syndromes |  |

1. Kroenke K, Spitzer RL, Williams JB. The PHQ-9: Validity of a brief depression severity measure. Journal of General Internal Medicine. 2001;16(9):606-13.

2. Young RC, Biggs JT, Ziegler VE, Meyer DA. A rating scale for mania: reliability, validity and sensitivity. British Journal of Psychiatry. 1978;133:429-35.

3. Angst J, Adolfsson R, Benazzi F, Gamma A, Hantouche E, Meyer TD, et al. The HCL-32: Towards a self-assessment tool for hypomanic symptoms in outpatients. Journal of Affective Disorders. 2005;88(2):217-33.

4. Spitzer RL, Kroenke K, Williams JB, Löwe B. A brief measure for assessing generalized anxiety disorder: the GAD-7. Archives of Internal Medicine. 2006;166(10):1092-7.

5. Heimberg RG, Horner K, Juster H, Safren S, Brown E, Schneier F, et al. Psychometric properties of the Liebowitz social anxiety scale. Psychological Medicine. 1999;29(1):199-212.

6. Goodman WK, Price LH, Rasmussen SA, Mazure C, Fleischmann RL, Hill CL, et al. The Yale-Brown Obsessive Compulsive Scale. I. Development, use, and reliability. Archive of General Psychiatry. 1989;46(11):1006-11.

7. Capra C, Kavanagh DJ, Hides L, Scott J. Brief screening for psychosis-like experiences. Schizophrenia Research. 2013;149(1-3):104-7.

8. Loewy RL, Pearson R, Vinogradov S, Bearden CE, Cannon TD. Psychosis risk screening with the Prodromal Questionnaire—brief version (PQ-B). Schizophrenia Research. 2011;129(1):42-6.

**Table S2. The 29 scales with 300 items were included as predicting features of general psychopathology and aggression with references.**

| **Domain** | **Scales** | **Label** | **N Items** | **Details** |  |
| --- | --- | --- | --- | --- | --- |
| **Demographics** | Age | Age | 1 |  |  |
|  | Biological Sex | Sex | 1 |  |  |
|  | Year of Education | Edu | 1 |  |  |
|  | Body Mass Index (BMI) (1) | BMI | 1 |  |  |
| **Cognition** | Digit Span WAIS III (2) | DS | 24 | Measure simple attention and working memory |  |
|  | Verbal Fluency: Animal Naming | VF | 1 |  |  |
|  | Information Subtest WAIS III (2) | Inf | 24 | Measure one’s ability to acquire, retain and retrieve information |  |
|  | Trail Making Test (3) | TMT | 3 | Measure visual attention and task switching |  |
|  | The Stroop Color and Word Test (4) | Stroop | 3 | Assess the ability to inhibit cognitive interference |  |
|  | Pattern Glare Test (5) | PG | 3 | Measure visual stress and visual perceptual distortions |  |
| **Lifestyle** | Insomnia Severity Survey (6) | ISS | 5 | Assess the severity of both nighttime and daytime components of insomnia |  |
|  | Pittsburgh Sleep Quality Index (7) | PSQI | 10 | Assess sleep quality and disturbances |  |
|  | Epworth Sleepiness Scale (8) | ESS | 8 | Measure daytime sleepiness |  |
|  | International Physical Activity Questionnaire (9) | IPAQ | 7 | Measure physical activity and inactivity |  |
|  | Alcohol Use Disorders Identification Test (10) | AUDIT | 10 | Measure hazardous and harmful alcohol consumption |  |
| **General Wellbeing** | 12-item Short Form Health Survey (11) | SF12 | 12 | Measure health-related quality of life |  |
|  | Social and Occupational Functioning Assessment Scale (12) | SOFAS | 1 | Assess social and occupational functioning |  |
|  | WHO Well-being Index (13) | WHO5 | 5 | Assess subjective psychological well-being |  |
| **Psychological Constructs** | Big Five Inventory (14-16) | BFI | 44 | Measure the Big Five personality dimensions |  |
|  | Rosenberg Self Esteem Scale (17) | SES | 10 | Measure of self-esteem |  |
|  | Procrastination Scale (18) | Procras | 20 | Measure procrastination behavior |  |
|  | Connor–Davidson Resilience Scale (19) | CDRISC | 10 | Assess resilience |  |
|  | Barratt Impulsiveness Scale (20) | BIS | 30 | Assess the personality/behavioral construct of impulsiveness |  |
|  | Goal Commitment Questionnaire (21) | GCQ | 9 | Measure goal commitment |  |
|  | Prosocial Items from the Primary Prevention Awareness, Attitudes and Usage Scale (22) | PPAAUS | 5 | Measure prosocial behavior |  |
|  | Future Outlook Inventory (23) | FOI | 14 | Measure one’s inclination to recognize and consider future events or consequences in everyday life |  |
|  | Youth Materialism Scale (24) | YMS | 10 | Measure materialism among youth |  |
|  | UCLA Loneliness Scale (25) | UCLA | 20 | Measure one's subjective feelings of loneliness as well as feelings of social isolation |  |
|  | Brief Measures of Sensation Seeking (26) | BSS | 8 | Measure sensation seeking |  |

1. Keys A, Fidanza F, Karvonen MJ, Kimura N, Taylor HL. Indices of relative weight and obesity. Journal of Chronic Diseases. 1972;25(6-7):329-43.

2. Wechsler DPC. WAIS-III: Administration and scoring manual: Wechsler Adult Intelligence Scale. [San Antonio, Tex]: Psychological Corporation; 1997.

3. Strauss E, Sherman EM, Spreen O. A compendium of neuropsychological tests: Administration, norms, and commentary: American Chemical Society; 2006.

4. Scarpina F, Tagini S. The Stroop Color and Word Test. Frontiers in Psychology. 2017;8.

5. Wilkins A, Evans B. Pattern glare test instructions. IOO Sales Ltd, London. 2001.

6. Morin CM, Belleville G, Bélanger L, Ivers H. The Insomnia Severity Index: Psychometric indicators to detect insomnia cases and evaluate treatment response. Sleep. 2011;34(5):601-8.

7. Buysse DJ, Reynolds CF, 3rd, Monk TH, Berman SR, Kupfer DJ. The Pittsburgh Sleep Quality Index: A new instrument for psychiatric practice and research. Psychiatry Research 1989;28(2):193-213.

8. Johns MW. A new method for measuring daytime sleepiness: the Epworth sleepiness scale. Sleep. 1991;14(6):540-5.

9. Craig CL, Marshall AL, Sjöström M, Bauman AE, Booth ML, Ainsworth BE, et al. International physical activity questionnaire: 12-country reliability and validity. Medicine & Science in Sports & Exercise. 2003;35(8):1381-95.

10. Saunders JB, Aasland OG, Babor TF, De La Fuente JR, Grant M. Development of the Alcohol Use Disorders Identification Test (AUDIT): WHO collaborative project on early detection of persons with harmful alcohol consumption-II. Addiction. 1993;88(6):791-804.

11. Ware J, Jr., Kosinski M, Keller SD. A 12-Item Short-Form Health Survey: Construction of scales and preliminary tests of reliability and validity. Med Care. 1996;34(3):220-33.

12. Rybarczyk B. Social and Occupational Functioning Assessment Scale (SOFAS). In: Kreutzer JS, DeLuca J, Caplan B, editors. Encyclopedia of Clinical Neuropsychology. New York, NY: Springer New York; 2011. p. 2313.

13. Topp CW, Østergaard SD, Søndergaard S, Bech P. The WHO-5 Well-Being Index: A Systematic Review of the Literature. Psychotherapy and Psychosomatics. 2015;84(3):167-76.

14. Benet-Martínez V, John OP. Los Cinco Grandes across cultures and ethnic groups: Multitrait-multimethod analyses of the Big Five in Spanish and English. Journal of Personality and Social Psychology. 1998;75(3):729.

15. John OP, Donahue EM, Kentle RL. Big five inventory. Journal of Personality and Social Psychology. 1991.

16. John OP, Naumann LP, Soto CJ. Paradigm shift to the integrative Big Five trait taxonomy: History, Measurement, and Conceptual Issues. 2008.

17. Rosenberg M. Rosenberg self-esteem scale. Journal of Religion and Health. 1965.

18. Lay CH. At last, my research article on procrastination. Journal of Research in Personality. 1986;20(4):474-95.

19. Connor KM, Davidson JR. Development of a new resilience scale: The Connor‐Davidson resilience scale (CD‐RISC). Depression and Anxiety. 2003;18(2):76-82.

20. Barratt ES, Monahan J, Steadman H. Impulsiveness and aggression. Violence and Mental Disorder: Developments in Risk Assessment. 1994;10:61-79.

21. Hollenbeck JR, Williams CR, Klein HJ. An empirical examination of the antecedents of commitment to difficult goals. Journal of Applied Psychology. 1989;74(1):18.

22. Carlo G, Randall BA. The development of a measure of prosocial behaviors for late adolescents. Journal of Youth and Adolescence. 2002;31(1):31-44.

23. Gunn RL, Pearman HE. An analysis of the future outlook of hospitalized psychiatric patients. Journal of Clinical Psychology. 1970;26(1):99-103.

24. Goldberg ME, Gorn GJ, Peracchio LA, Bamossy G. Understanding materialism among youth. Journal of Consumer Psychology. 2003;13(3):278-88.

25. Russell DW. UCLA Loneliness Scale (Version 3): Reliability, validity, and factor structure. Journal of Personality Assessment. 1996;66(1):20-40.

26. Hoyle RH, Stephenson MT, Palmgreen P, Lorch EP, Donohew RL. Reliability and validity of a brief measure of sensation seeking. Personality and Individual Differences. 2002;32(3):401-14.

**Table S3. No difference between the original dataset and complete samples was found.**


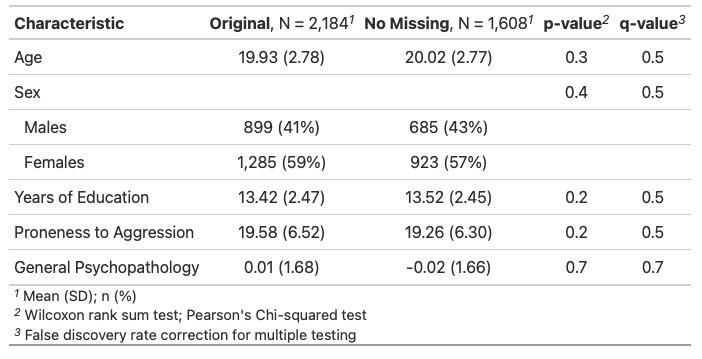


**Figure S1. Results of the PCA analysis on symptom levels of psychopathology.** A. Scree plot shows the explained variance of each PCA component. B. Loadings of each PCA component.

**
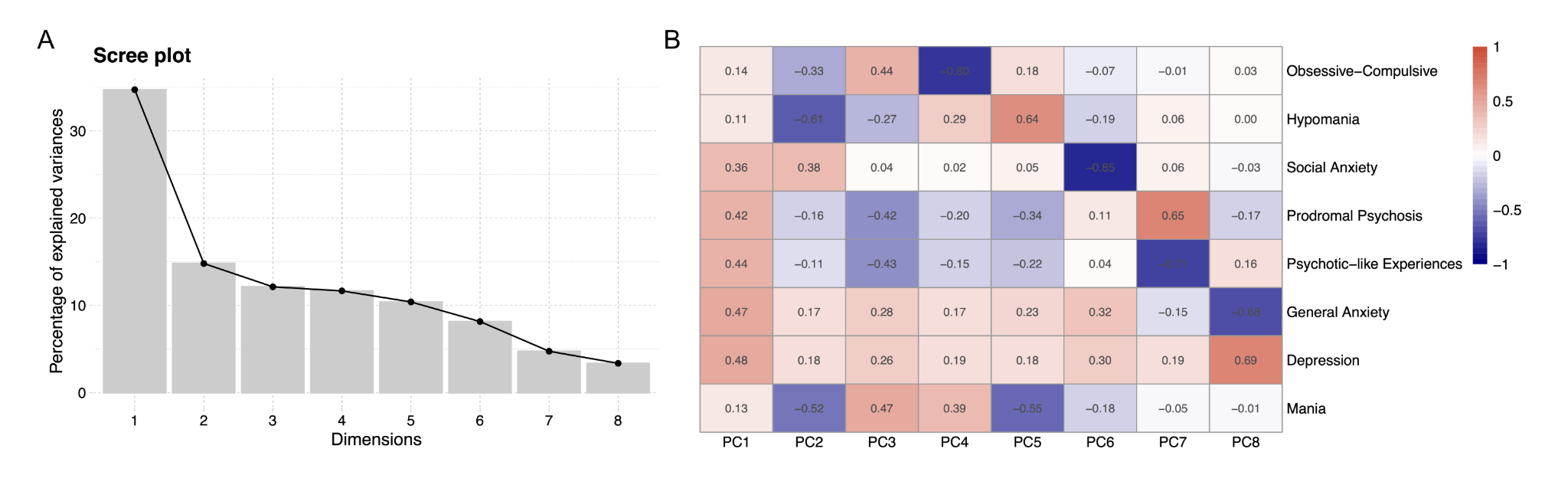
**

**Figure S2. Performance for predicting general psychopathology and proneness to aggression using Elastic Net.**

**
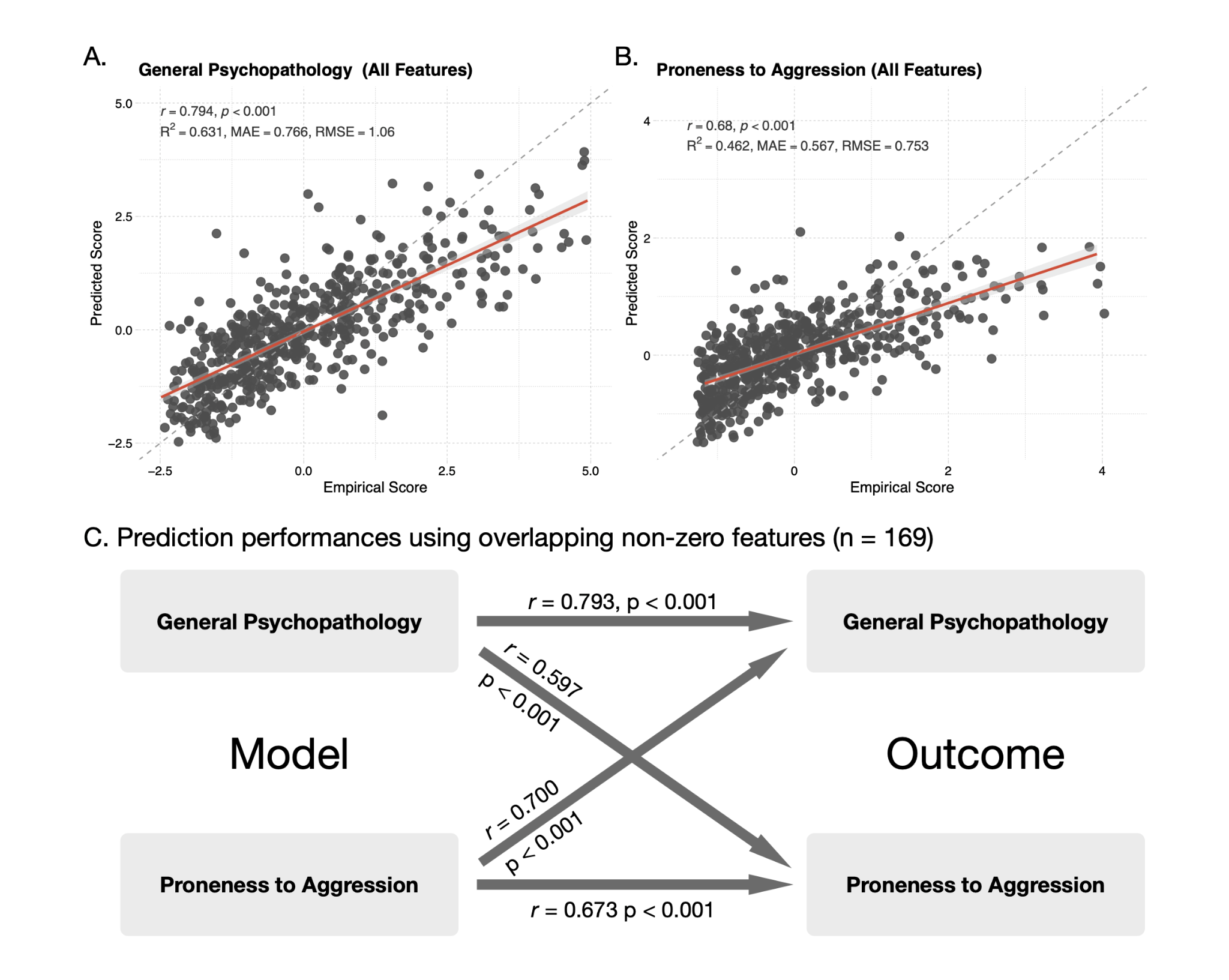
**

**Figure S3. Parallel analysis suggested 18 latent factors over 102 overlapping features.**

**
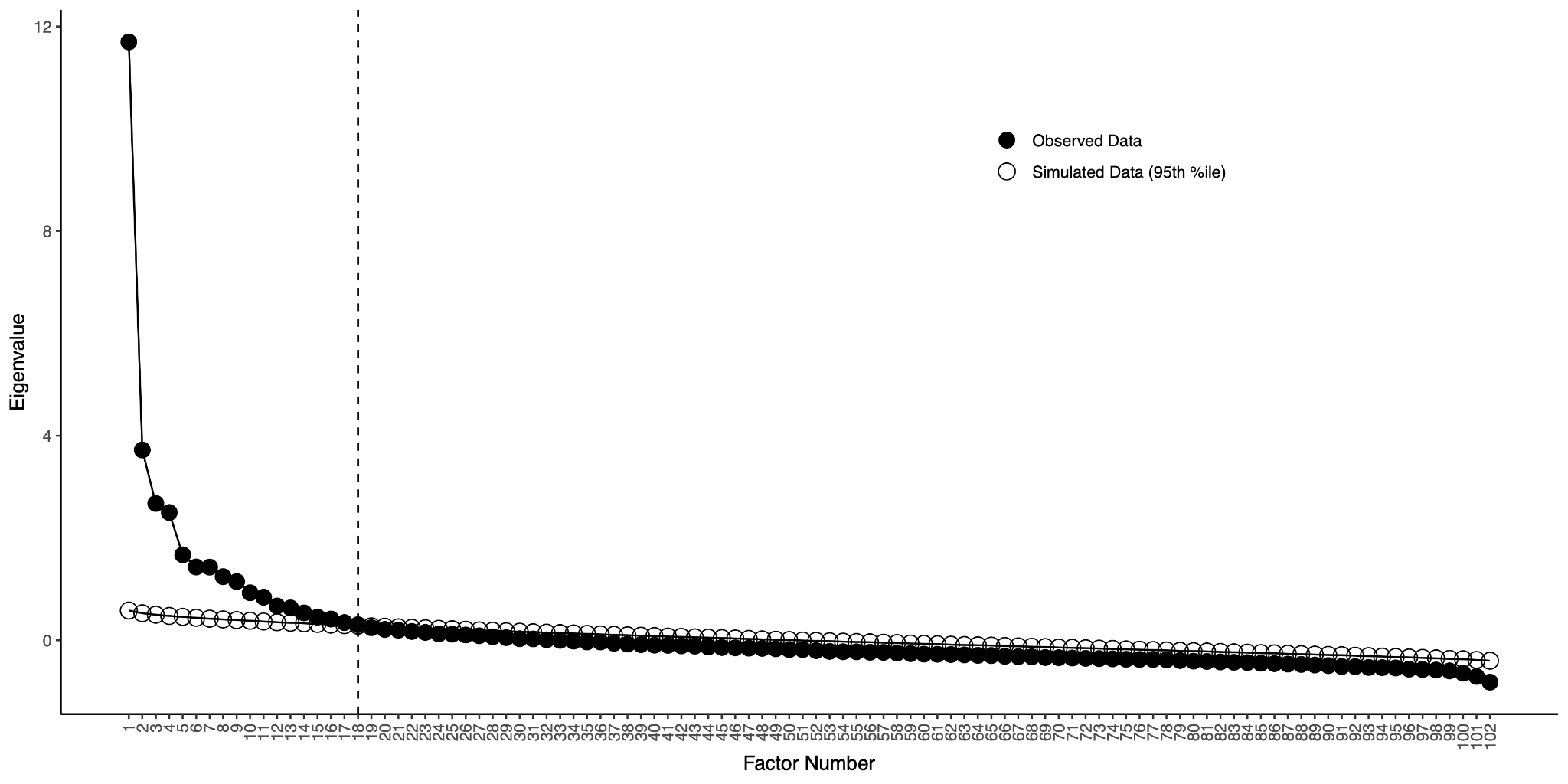
**

**Figure S4. Results of factor analysis on the overlapping features of general psychopathology and proneness to aggression. A.** Correlations between 18 factor pairs. **B.** Heatmap of loadings of each factor. Labeling of each factor was based on the top loadings (highlighted in red or blue) of each factor. See Table S2 for scale abbreviations.


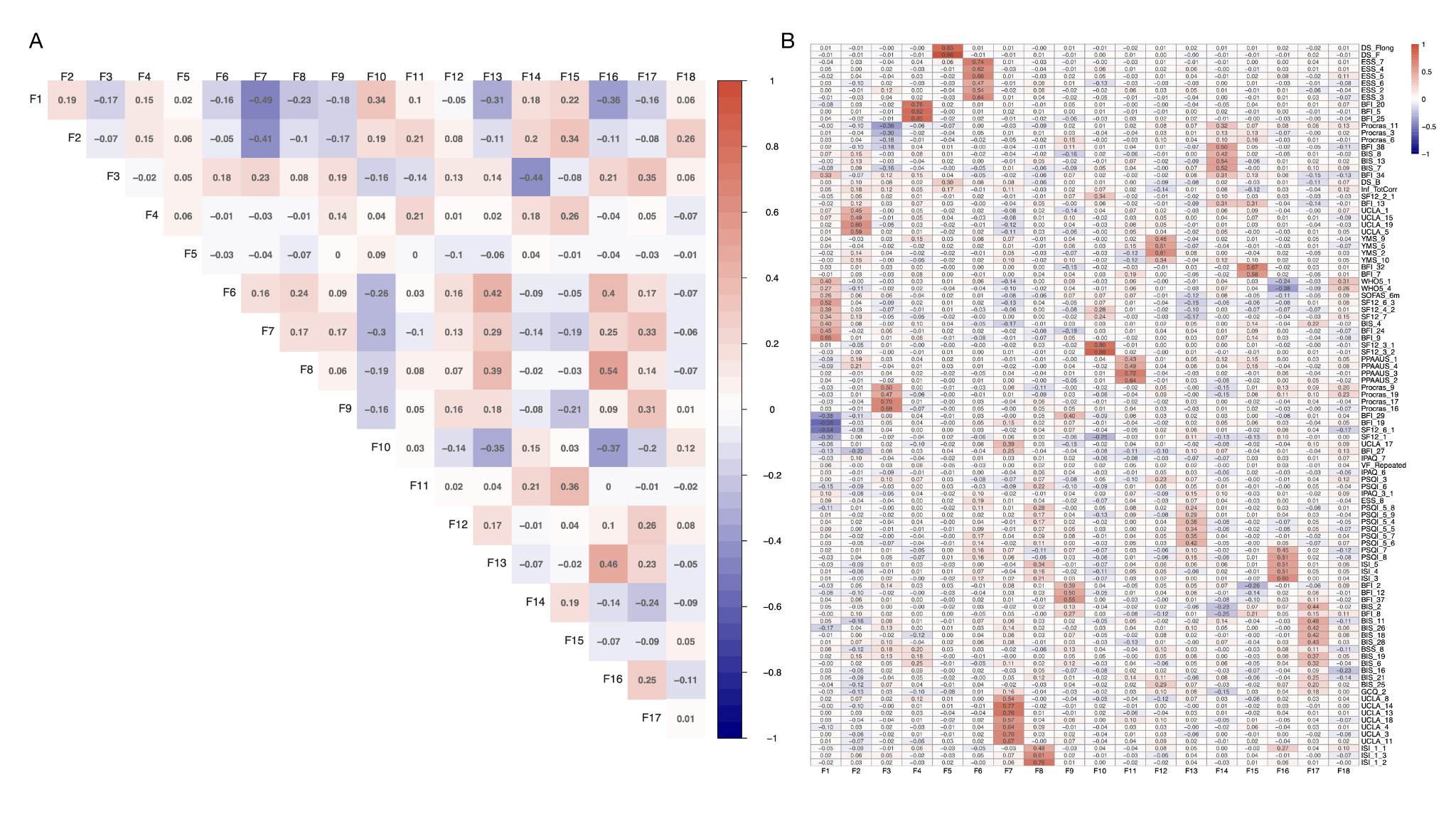


Figure S5. Histogram plots of transformed input variables of the GGM.

**
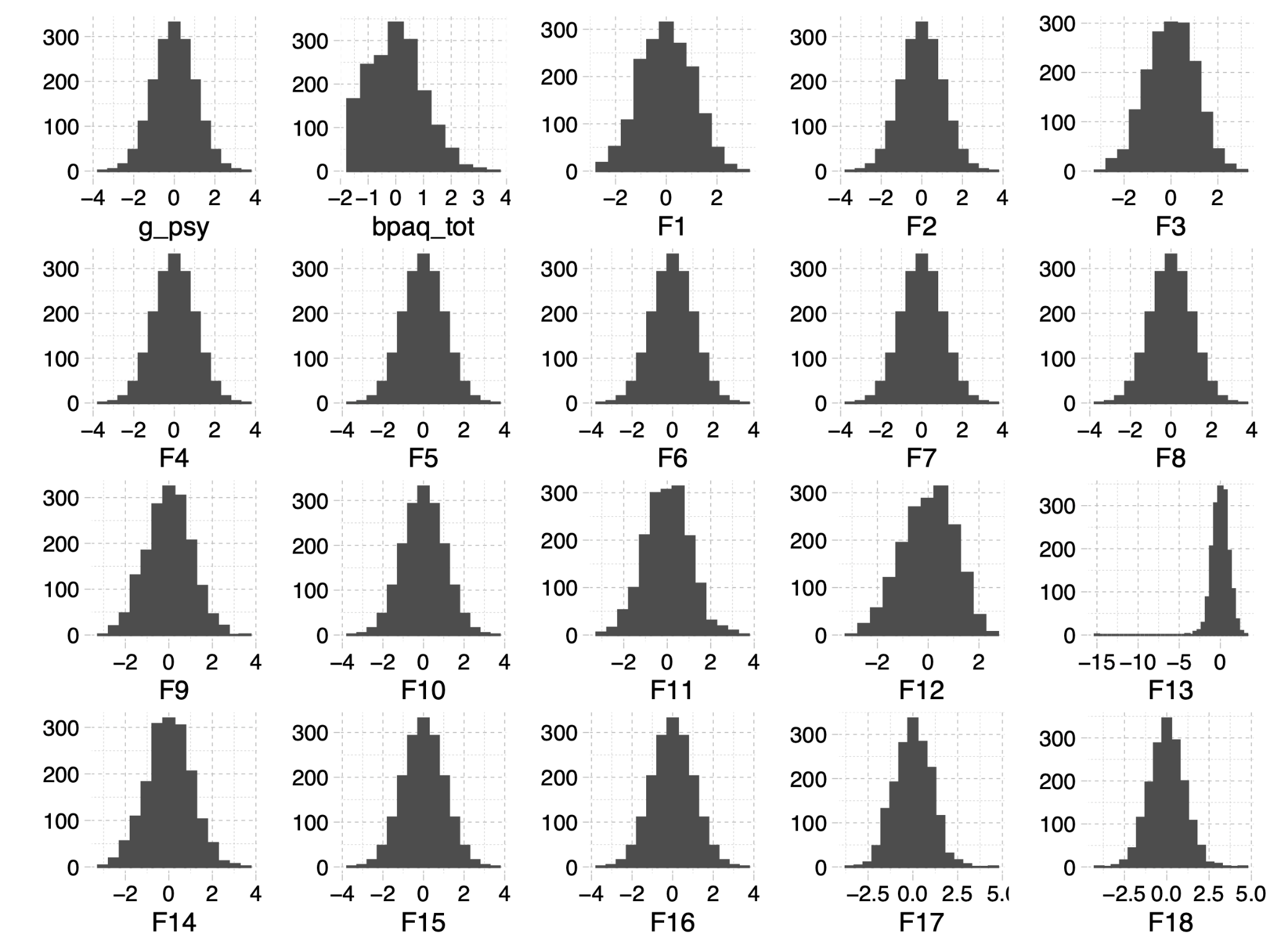
**

**Figure S6. Node centrality indices of the GGM network.**


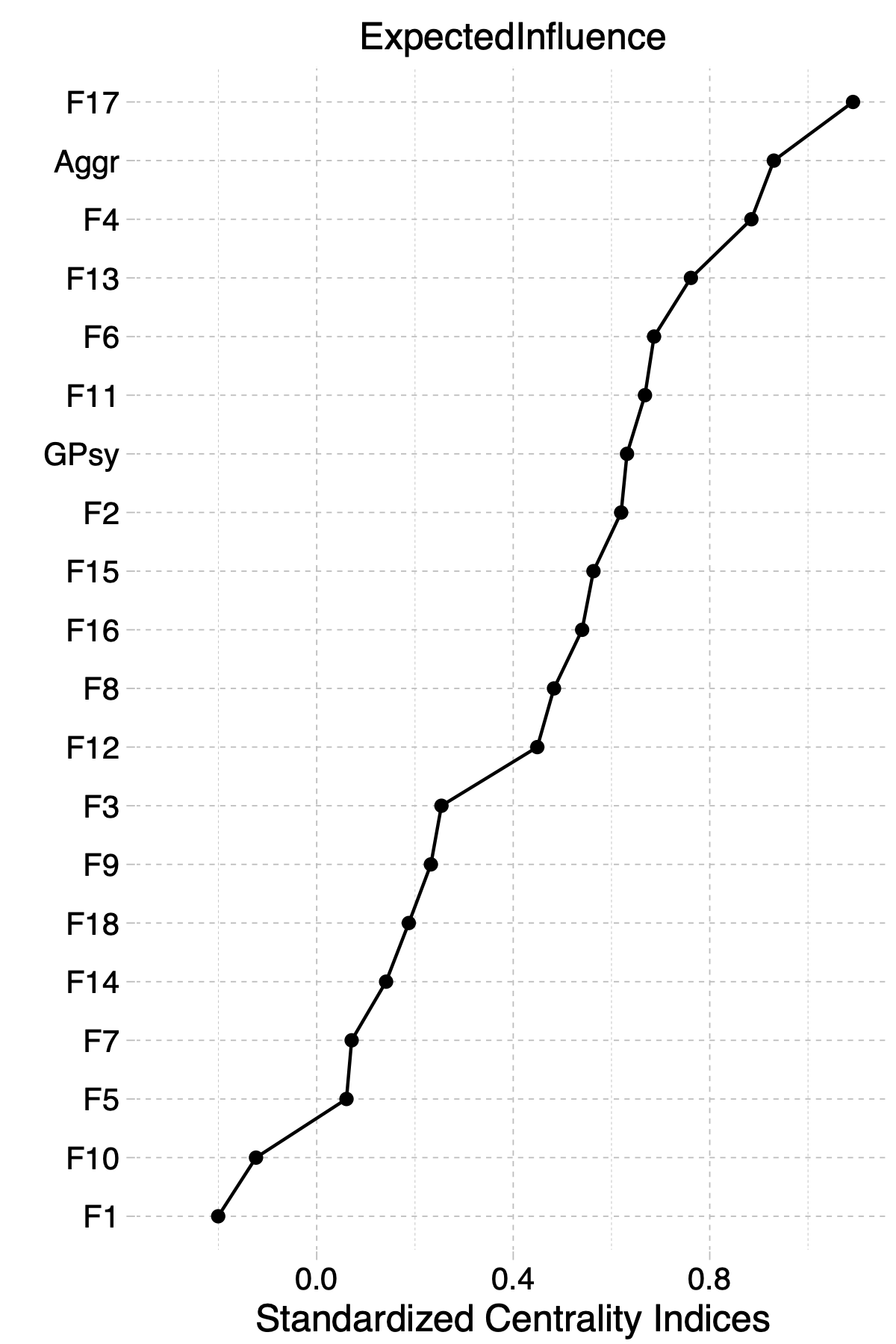


**Figure S7. Stability of edge strengths.**

**
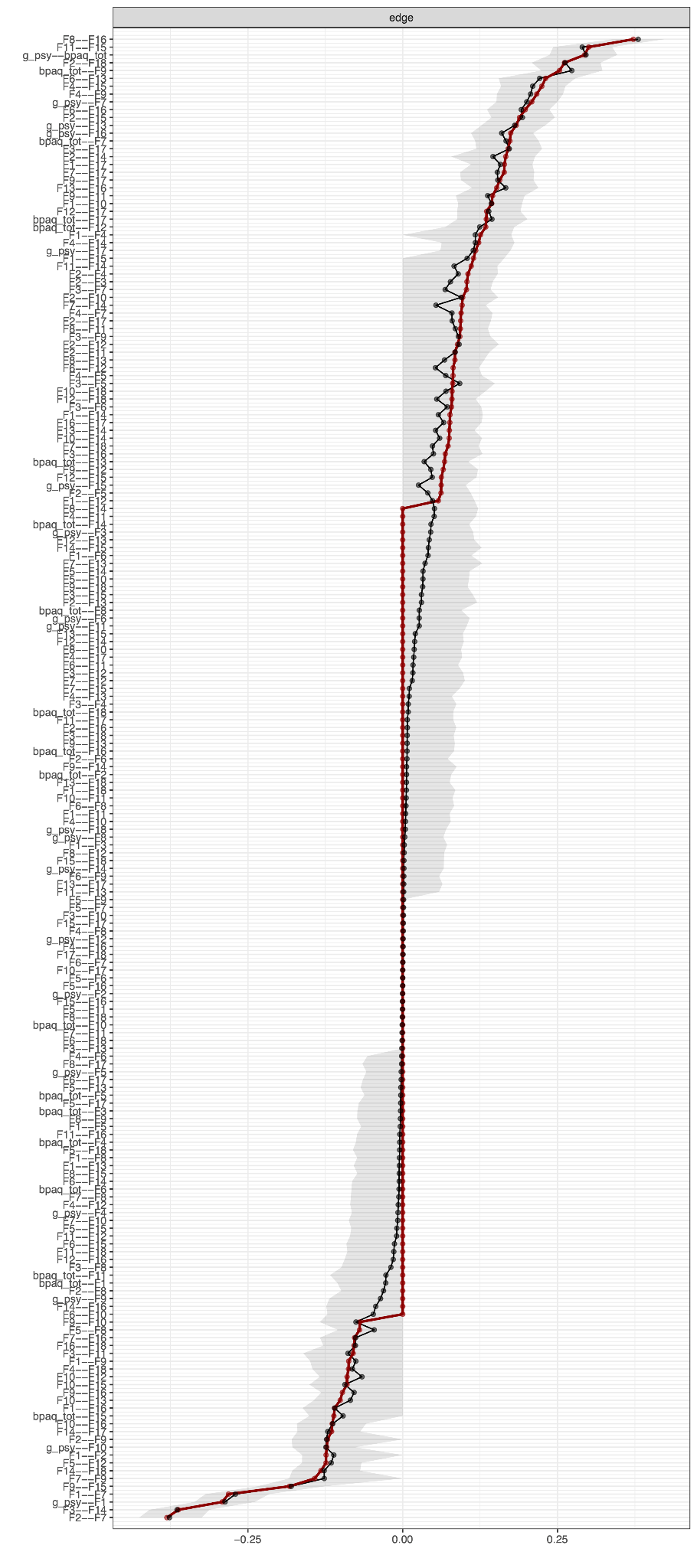
**

**Figure S8. Stability of centrality index.** Strength centrality estimate was stable, with a centrality stability coefficient of 0.85, indicating that 75% of the data could be dropped to retain with 95% certainty a correlation of 0.7 with the original dataset.

**
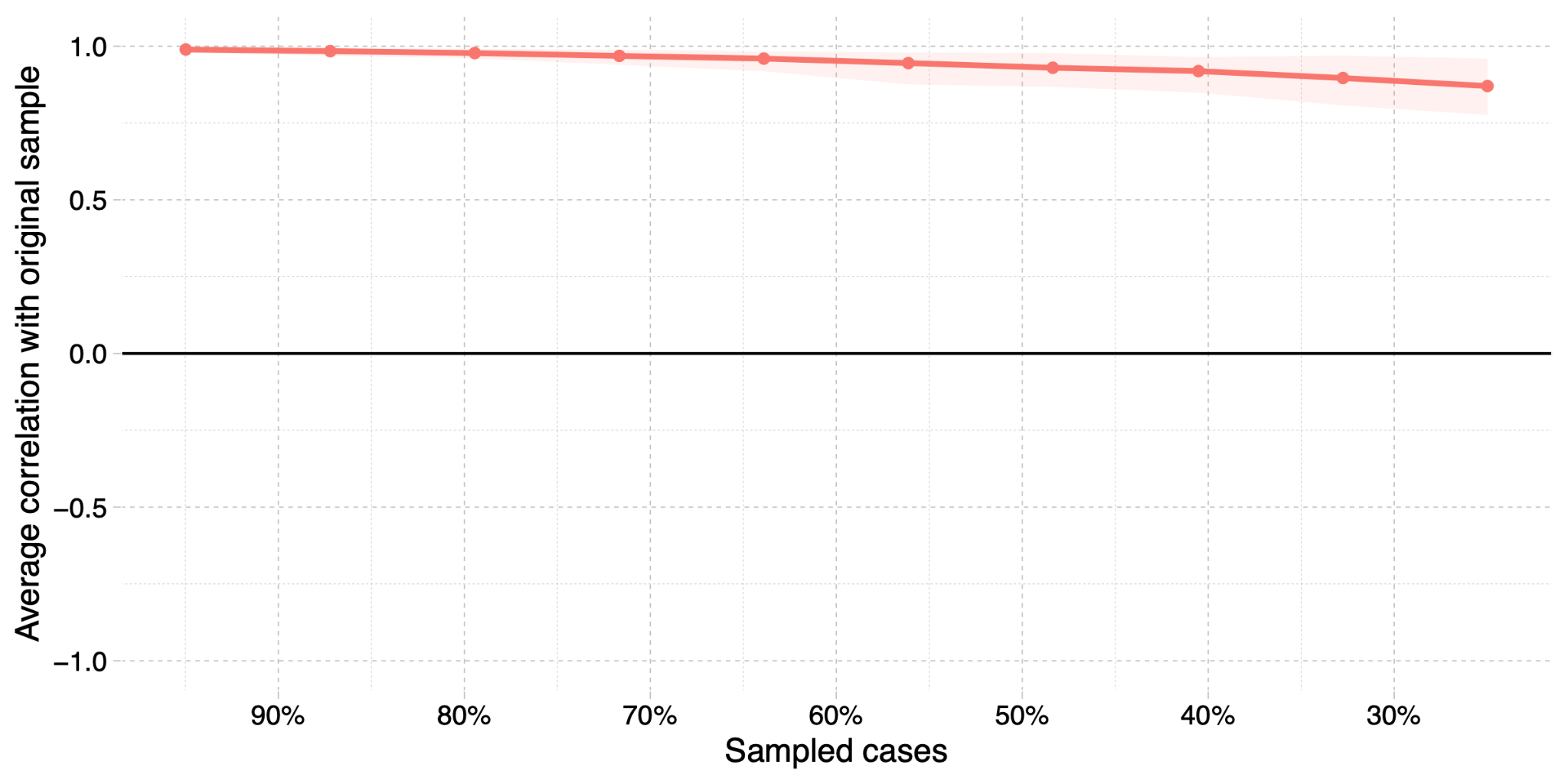
**
